# Strain-Dependent Porcine Circovirus Type 2 (PCV2) Entry and Replication in T-Lymphoblasts

**DOI:** 10.3390/v11090813

**Published:** 2019-09-02

**Authors:** Ruifang Wei, Nicolaas Van Renne, Hans J. Nauwynck

**Affiliations:** Laboratory of Virology, Faculty of Veterinary Medicine, Ghent University, Salisburylaan 133, B-9820 Merelbeke, Belgium

**Keywords:** porcine circovirus type 2, Stoon1010, 1121, T-lymphoblasts, replication, binding, entry, disassembly, evolution

## Abstract

Porcine circovirus type 2 (PCV2) is the etiological agent of PCV2-associated diseases (PCVAD). PCV2 targets lymphoblasts, and pigs suffering from PCVAD display lymphocyte depletion in lymphoid tissues. PCV2 infection of lymphoblasts has not been studied. Here, the replication cycle of PCV2 (abortion strain 1121 and PMWS strain Stoon1010) in T-lymphoblasts was examined. The expression of Rep and Cap were found for both viral strains, while progeny virus was detected for Stoon1010 but not for 1121. PCV2 attached to 11–26% (1121-Stoon1010) of the T-lymphoblasts while 2.6–12.7% of cells showed virus internalization. Chondroitin sulfate (CS) was present on 25% of T-lymphoblasts, and colocalized with PCV2 on 31–32% of the PCV2+ cells. Enzymatic removal of CS reduced PCV2 infection. PCV2 infection was decreased by chlorpromazine, cytochalasin D and *Clostridium difficile* toxin B for both viral strains and by amiloride for 1121 but not for Stoon1010. Inhibiting either endosome acidification or serine proteases strongly reduced PCV2 infection. Three-dimensional analysis of Cap structure demonstrated a better Cap-nucleic acid affinity for Stoon1010 than for 1121. Taken together, PCV2 binds to T-lymphoblasts partially via CS, enters via clathrin-mediated endocytosis, and disassembles under functions of a pH-drop and serine proteases. Strain Stoon1010 displayed an enhanced viral binding, a specific receptor-mediated endocytosis, an increased Cap-nucleic acid affinity, and a more productive infection in T-lymphoblasts than 1121 did, indicating an evolution from 1121 to Stoon1010.

## 1. Introduction

Porcine circovirus type 2 (PCV2) belongs to the genus *Circovirus* of the family *Circoviridae* [1]. It is a globally recognized viral pathogen of great importance in the swine industry [2]. The icosahedral-shaped and non-enveloped PCV2 virion consists of 60 capsomeres and a single-stranded circular DNA genome of 1768 bases [3,4]. PCV2 DNA is ubiquitously detected in the environment, for example, in water samples in Brazil, farm air in Canada and house flies in UK [5,6,7]. It is also identified in a variety of non-porcine species, such as rats, calves, minks and foxes [8,9,10,11]. Unexpectedly, PCV2 DNA was able to persist in a human RD cell line (ATCC CCL-136), although PCV2 gene replication and protein expression were not observed in vitro [12].

Within the PCV2 genome, two major open reading frames (ORFs) are well-identified. ORF1 encodes the replicase protein (Rep) which is indispensable for the rolling-circle replication of PCV2 genome; ORF2 encodes the capsid protein (Cap) which is the major immunogenic protein of PCV2 [13,14,15]. Although being small-sized and simple-structured, PCV2 is the major cause of the ravaging postweaning multisystemic wasting syndrome (PMWS) and many other syndromes which are generally regarded as porcine circovirus associated diseases (PCVAD) [16,17,18]. In diseased pigs, PCV2 is consistently found in cells of the monocyte/macrophage lineage [19,20,21,22,23]. Its entry into the monocytic 3D4/31 cells in vitro initiates with binding to heparan sulfate (HS) and chondroitin sulfate B (CS-B) glycosaminoglycans (GAGs) on the cell surface before entering the cell via clathrin-mediated endocytosis [24,25]. The same pathway is used to mediate the internalization of PCV2 to dendritic cells and primary monocytes [26,27]. After entry, PCV2 is either disassembled in an acid environment by serine proteases in the monocytic 3D4/31 cells or partially disintegrated in primary monocytes [25,27]. The incapability of a full degradation of capsids by primary monocytes may in part explain the frequent detection of PCV2 in these cells. PCV2 antigens and/or nucleic acids were also found in other cell types including lymphocytes, hepatocytes, enterocytes, renal and alveolar epithelial cells, vascular endothelial cells, smooth muscle cells and fibroblasts [28]. Up to now, PCV2 entry into epithelial cells has been characterized via an actin- and small-GTPase-dependent pathway and needs a neutral environment for virion disassembly aided by serine proteases [29].

In contrast to the non-productive infection of PCV2 in cells of the monocyte/macrophage lineage, lymphoblasts are fully susceptible targets of PCV2 [19,30,31]. The larger the number of blasts, the faster and higher the primary replication of PCV2 in its host [31]. The degree of PCV2 replication in lymphoblasts might be related with the severity of the diseases. Recent work demonstrated that in vitro generated porcine T-lymphoblasts support PCV2 replication [32]. This provides a new and valuable tool to study the pathogenesis of PCV2 in one of its real targets. However, the detailed mechanism of PCV2 entry and replication in the T-lymphoblasts has not been studied yet. Here, we investigated the replication kinetics of PCV2 (abortion strain 1121 and PMWS strain Stoon1010) in porcine T-lymphoblasts by doing time-course experiments. With confocal microscopy and chemical inhibitors, PCV2 binding, entry and disassembly of PCV2 in T-lymphoblasts were visualized and analyzed. The difference between strains 1121 and Stoon1010 was further revealed in amino-acid and structural levels.

## 2. Materials and Methods 

### 2.1. Generation of Porcine T-Lymphoblasts In Vitro

**Figure 1:**
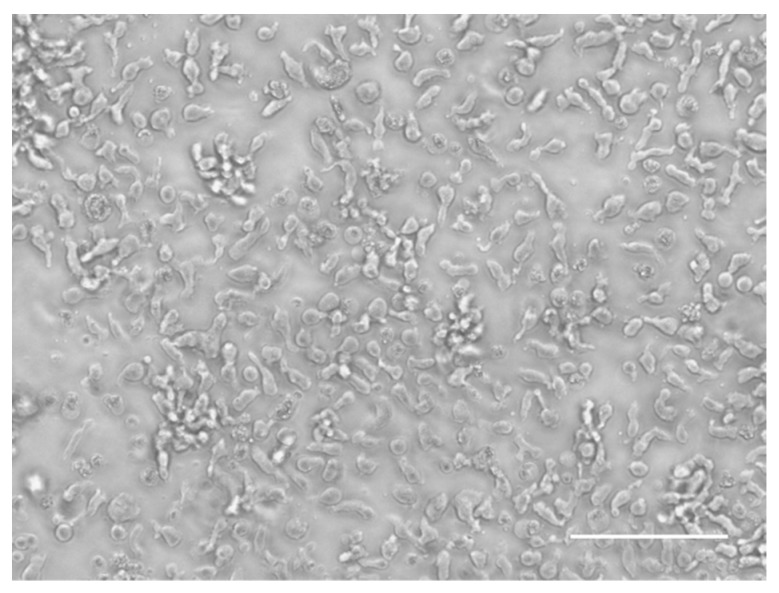
Morphology of in vitro generated T-lymphoblasts. Scale bar = 100 µm.

Porcine T-lymphoblasts were generated from a 10-week-old healthy pig as described previously [32]. Briefly, PBMCs were isolated by density gradient centrifugation on Ficoll–Paque, resuspended in RPMI supplemented with 5% fetal calf serum (FCS, Gibco, Paisley, UK) and antibiotics and cultured for 18 h at 37 °C [27]. Non-adhering lymphocytes were pelleted and resuspended in leukocyte medium (RPMI, supplemented with 10% FCS, antibiotics, 100 µg/mL Na-pyruvate and 100 mM non-essential amino acids) in the presence of 5 µg/mL concanavalin A (ConA, Sigma-Aldrich, St. Louis, MO, USA) and 50 µM β-mercaptoethanol (Gibco). Three days later, cells were pelleted, washed with RPMI and resuspended in culture medium based on leukocyte medium supplemented with 100 U/mL human recombinant interleukin-2 (IL-2, NIH) and 50 µM β-mercaptoethanol. Cells were passaged every four days and the cell morphology was examined using light microscopy (Olympus, Hamburg, Germany) (Figure 1).

### 2.2. Virus Preparation and Particle Quantification 

PCV2a strain 1121 that was isolated from aborted fetuses in Canada, and strain Stoon1010 that was isolated from PMWS-affected piglets in Canada were used in this study [33,34]. Virus stocks, propagated in PK-15 cells (a kind gift from Gordon Allan, Queen’s University Belfast) with a titer of 10^5^ TCID_50_/mL were used in infection experiments at 0.05 TCID_50_/cell. For attachment and internalization experiments, virus stocks were filtered with 0.45 µm filters to remove cell components and large aggregates of virus particles, followed by a quantification of virus particles as described earlier [27]. To allow a clear visualization of virus attachment/internalization, 10^10^ virus particles/well were used in these experiments.

### 2.3. PCV2 Peplication Kinetics in T-Lymphoblasts

T-lymphoblasts were inoculated with PCV2 (strains 1121 and Stoon1010) at 0.05 TCID_50_/cell at 37 °C for 1 h. After inoculation, cells were washed twice, resuspended in fresh culture medium and seeded 1 mL/well in 24-well cell culture plates at a concentration of 3 × 10^5^ cells/mL. At 0 h, 12 h, 24 h, 36 h, 48 h and 72 h, the supernatant and cells of inoculated cultures were collected. Supernatant fluids were titrated by immunoperoxidase monolayer assay (IPMA) and the number of viral genome copies was quantified by qPCR as described elsewhere [27,35]. Cells were smeared onto microscope slides, followed by a fixation of cells with 4% paraformaldehyde (PF) at room temperature (RT) for 10 min and a permeabilization with 0.1% TritonX-100 at RT for 2 min. To visualize the expression of the non-structural Rep protein and the structural Cap protein, a double immunofluorescence staining was performed. Rep was stained with mouse monoclonal antibody (mAb) F210 (1:100, IgG1; a kind gift from Gordon Allan, Queen’s University Belfast) [36], followed by an Alexa Fluor 594-conjugated goat-anti-mouse IgG1 antibody (1:200; Life Technologies, Carlsbad, CA, USA), while Cap was visualized with our home-made mouse mAb 94H8 (1:100, IgG2a) [37], in combination with an Alexa Fluor 488-conjugated goat-anti-mouse IgG2a antibody (1:200; Life Technologies). The cell nuclei were counterstained with Hoechst 33,342 (Molecular Probes) for 10 min at RT. Afterwards, cells were mounted with a glycerol solution containing 1,4-diazabicyclo (2.2.2) octane (DABCO, Acros, Ghent, Belgium) anti-fading agent and were analyzed under a Leica TCS SPE laser-scanning spectral confocal microscope. For each sample, 10 random images were taken and the percentages of PCV2 Rep or Cap positive cells were calculated from these images.

### 2.4. PCV2 Attachment to T-Lymphoblasts

#### 2.4.1. Visualization of PCV2 Attachment

T-lymphoblasts (2 × 10^5^ cells) were incubated with 10^10^ PCV2 particles (strains 1121 and Stoon1010) at 4 °C for 1 h. After incubation, the virus was removed and cells were washed twice. Then, cells were smeared onto microscope slides and were fixed with 4% PF at RT for 10 min. A double immunofluorescence staining was carried out to visualize cells and virus particles. In detail, cells were stained with an mAb against CD3 (clone PPT3 supernatant, 1:5, IgG1), followed by an Alexa Fluor 594-conjugated goat-anti-mouse IgG1 antibody (1:200; Life Technologies), while the attached virus particles were stained with 94H8 as described in Section 2.3. The cell nuclei were stained with Hoechst 33342, after which samples were mounted and analyzed under the confocal microscope. For each sample, 10 random images were taken. The percentage of cells with attached virus particles were calculated from these images. The fluorescing area of the bound virus particles per cell was quantified with ImageJ.

#### 2.4.2. Determination of the Expression of GAGs on T-Lymphoblasts

T-lymphoblasts were smeared onto glass slides and fixed with 4% PF at RT for 10 min. To determine the expression of HS and CS, cells were stained respectively with a monoclonal mouse anti-HS antibody (10E4, 1:50; Ambsio, Abingdon, UK) or a monoclonal mouse anti-CS (CS-56, 1:50; Bio-rad, Temse, Belgium) followed by FITC-labelled goat anti-mouse IgM antibodies (1:100; Life Technologies). PK-15 cells, which are known for the expression of HS and CS, were stained in parallel as a positive control [24]. To determine the expression of keratan sulfate (KS) and hyaluronic acid (HA), cells were stained with a monoclonal mouse anti-KS antibody [MZ15, 1:10, deposited to the DSHB (the Developmental Studies Hybridoma Bank) by Watt, F.M. [38] or a monoclonal mouse anti-HA antibody (1:100; Cloud-Clone Corp, Katy, TX, USA.), followed by FITC-labelled goat anti-mouse IgG1 antibodies (1:200; Life Technologies). In parallel, the brain tissue which is known to express KS [39] and the lung tissue which is known to express HA [40], were used as positive controls. These tissues were archived samples stored in our lab. Cells with only the secondary antibody incubation served as a control. The cell nuclei were stained with Hoechst 33342. The expression of GAGs on T-lymphoblasts was examined by confocal microscopy. The percentage of positive cells was calculated from 10 randomly selected regions.

#### 2.4.3. Evaluation of PCV2 Particles Binding to CS-Expressed T-Lymphoblasts

The PCV2 binding assay was performed as described in Section 2.4.1. After fixation of the inoculated cells, a double immunofluorescence staining was performed to visualize bound virus particles and the expression of CS on T-lymphoblasts. CS was stained as mentioned above in Section 2.4.2, while the attached virus particles were stained with 94H8, followed by an Alexa Fluor 488-conjugated goat-anti-mouse IgG2a antibody. The cell nuclei were stained with Hoechst 33342, after which samples were mounted and analyzed under the confocal microscope. The percentage of cells showing virus binding with or without the expression of CS were examined (500 cells). 

#### 2.4.4. Effect of Enzymatic Treatment on PCV2 Infection in T-Lymphoblasts

For enzymatic removal of cell surface chondroitin sulfate, T-lymphoblasts were incubated with chondroitinase ABC (2 U/mL; Sigma) from *Proteus vulgaris* diluted in Dulbecco’s PBS (DPBS) for 1 h at 37 °C. The concentration of chondroitinase ABC used in this study did not decrease the cell viability (˃90%). After washing, untreated and enzyme-treated cells were inoculated with PCV2 1121 or Stoon1010 strains at 0.05 TCID_50_/cell for 1 h at 37 °C. Cells were washed and further cultured for 24 h at 37 °C until fixation and permeabilization. PCV2-infected cells were detected by immunofluorescence staining using our home-made mouse mAb against Cap (38C1, 1:50, IgG1) [37] and FITC-labelled goat anti-mouse IgG antibodies. The infection level in the treated group was expressed as the relative percentage to that of the control group (without enzyme treatment).

### 2.5. PCV2 Entry and Disassembly in T-Lymphoblasts

#### 2.5.1. Visualization of PCV2 Internalization

T-lymphoblasts (2 × 10^5^ cells) were incubated with 10^10^ PCV2 particles (strains 1121 and Stoon1010) at 37 °C for 1 h. Then, the virus was removed and cells were washed twice. Cell smears were fixed with 4% PF and permeabilized with 0.1% TritonX-100, after which cells and virus particles were stained as described in Section 2.4.1. The percentage of cells showing internalized virus particles were analyzed using confocal microscopy.

#### 2.5.2. Effect of Different Entry Inhibitors on PCV2 Infection in T-Lymphoblasts

T-lymphoblasts were pre-treated for 2 h at 37 °C with different concentrations of inhibitory compounds: amiloride (0, 0.25, 0.5 or 1 mM) dissolved in DMSO, methyl-β-cyclodextrin (mβCD; 0, 0.63 or 1.25 mM) dissolved in medium, chlorpromazine (0, 3.13, 6.25 or 12.5 µM) dissolved in PBS, cytochalasin D (0, 6.25, 12.5 or 25 µM) dissolved in DMSO and *Clostridium difficile* toxin B (0, 12.5, 25 or 50 ng/mL) dissolved in media. Cells were then inoculated with PCV2 strain 1121 or Stoon1010 at 0.05 TCID_50_/cell in the presence of the same inhibitors at 37 °C for 1 h. After washing, cells were incubated for 24 h in the absence of inhibitors. The active concentrations of inhibitors were optimized to ensure that the cell viability was always ˃75%. The percentage of Cap-positive cells was determined as described in Section 2.4.3. The level of PCV2 infection in the different inhibitor groups was expressed as relative percentage to that of the control group (without inhibitor).

#### 2.5.3. Effect of Acidotropic Agents on PCV2 Infection in T-Lymphoblasts

Cells were pre-incubated with different concentrations of ammonium chloride (0, 0.1, 1 or 10 mM) or chloroquine (0, 1, 10 or 50 µM) dissolved in medium for 2 h at 37 °C. Untreated and treated cells were then inoculated with PCV2 strain 1121 or Stoon1010 at 0.05 TCID_50_/cell in the presence of the drugs for 1 h. After washing, cells were further cultured in the presence of the drugs for 24 h at 37 °C. The concentrations of agents used in this study did not decrease the cell viability (˃90%). The relative infection percentage of the treated groups to the untreated group was determined as described in Section 2.4.3.

#### 2.5.4. Effect of AEBSF on PCV2 Infection in T-Lymphoblasts

Cells were pre-treated with non-toxic concentrations of 4-(2-aminoethyl) benzenesulfonyl fluoride (AEBSF; 0, 0.1, 0.2 mM) dissolved in medium for 1 h at 37 °C. Cells were then inoculated with PCV2 strain 1121 or Stoon1010 at 0.05 TCID_50_/cell in the presence of AEBSF at 37 °C for 1 h. After washing, cells were incubated in the presence of the drug for 12 h and further cultured in fresh medium without inhibitors until 24 h. The concentrations of AEBSF were optimized to ensure that the cell viability was always ˃75%. The relative infection percentage of the treated groups to the untreated group was determined as described in Section 2.4.3.

### 2.6. Toxicity Assays

To examine the toxicity of chemical inhibitors on T-lymphoblasts, cells were treated with chemical reagents for the same durations as in the infection assays. Cytotoxic effect on cells was assessed in triplicate by analyzing the ability to metabolize 3-(4,5-dimethylthiazol-2-yl)-2,5-diphenyltetrazolium bromide (MTT) [41]. Formazan crystals were solubilized 4 h after adding MTT [41].

### 2.7. Amino Acid and 3D Structural Analyses of PCV2 Cap Sequences from Strains 1121 and Stoon1010

PCV2 Cap sequences from strains 1121 (GeneBank accession #AJ293868) and Stoon1010 (GeneBank accession #AF055392) were downloaded from GeneBank and were aligned using the Clustal W method of the MegAlign program of the DNASTAR version 7.0. The accurate 3D structures of PCV2 Cap protein from strains 1121 and Stoon1010 were predicted using iterative threading assembly refinement (I-TASSER) server (https://zhanglab.ccmb.med.umich.edu/I-TASSER/) [42,43,44]. The structures of Cap protein were displayed with PyMOL software (South San Francisco, CA, US). 

### 2.8. Statistical Analysis

Data were analyzed with GraphPad Prism 5 software (GraphPad Software, La Jolla, CA, USA). For the kinetics study, differences between two strains were assessed by two-way ANOVA. For virus attachment/internalization study, differences between two strains were revealed by the Student’s *t*-test. All results represent means ± SD. Results with a *p* value of <0.05 were considered statistically significant.

## 3. Results

### 3.1. The In Vitro Generated T-Lymphoblasts Support PCV2 Replication

**Figure 2:**
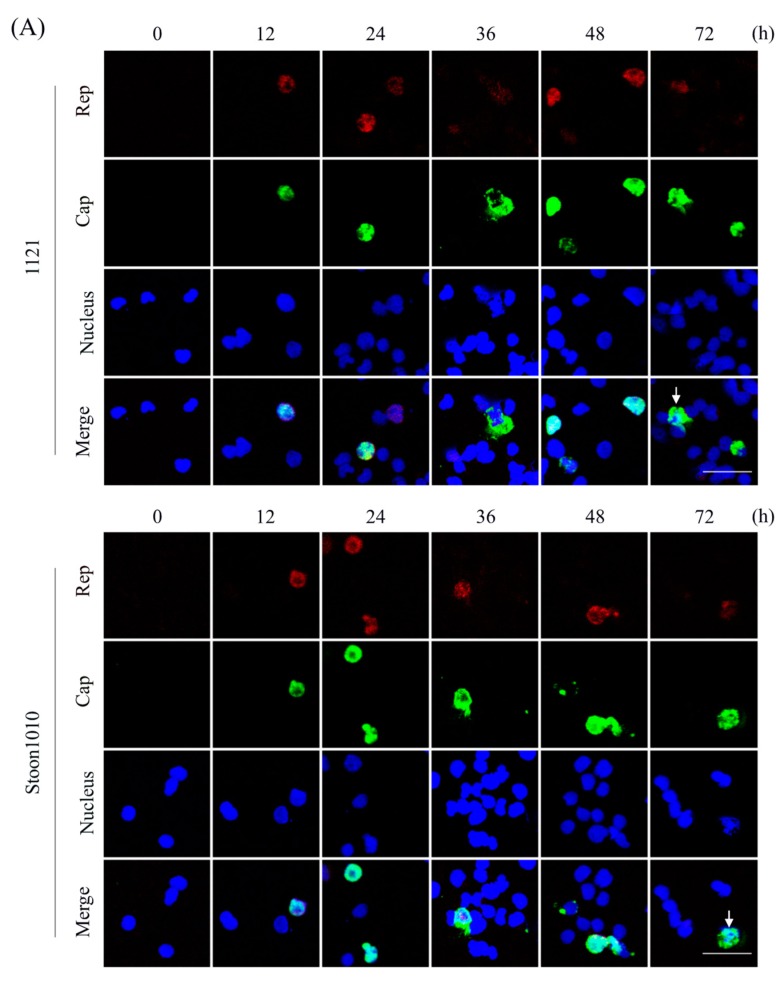

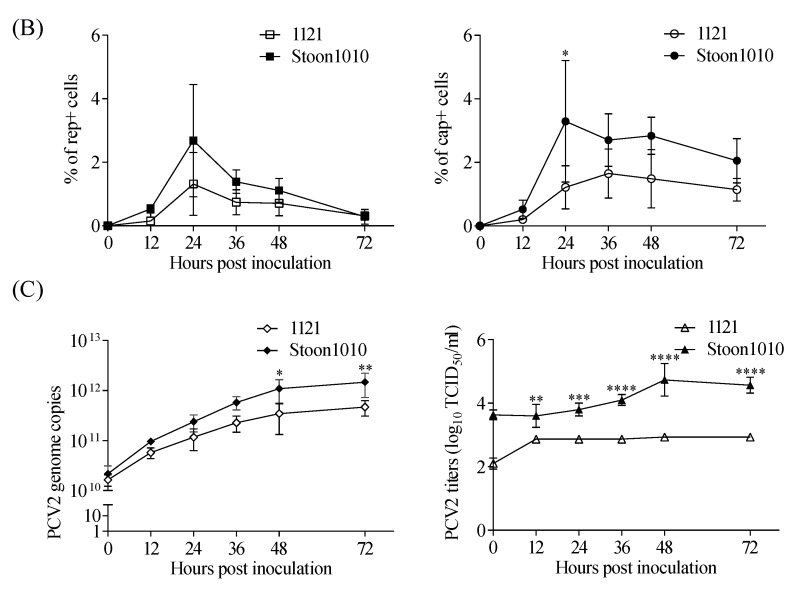
Porcine circovirus type 2 (PCV2) replication kinetics in T-lymphoblasts. Cells were inoculated with PCV2 strain 1121 or Stoon1010 at 0.05 TCID_50_/cell at 37 °C for 1 h. Afterwards, the virus was removed and cells were cultured until the indicated timepoints. At each timepoint, cells were fixed and stained for the expression of PCV2 replicase (Rep) and capsid (Cap) proteins, while the culture supernatant was collected and examined for genome copies by qPCR and virus titration. (**A**) Confocal images showing the expression kinetics of Rep and Cap, with strain 1121 shown on top and strain Stoon1010 at the bottom. White arrows indicate irregularly shaped cells with intense PCV2 antigen expression and deformed nucleus. Scale bar= 50 µm. (**B**) The percentages of Rep+ cells and Cap+ cells were quantified for strain 1121 and Stoon1010, respectively. (**C**) Evolution of PCV2 genome copies and titers in the supernatant of T-lymphoblast cultures inoculated with strains 1121 or Stoon1010. Data represent mean ± SD of triplicate assays. * *p* < 0.05; ** *p* < 0.01; *** *p* < 0.001; **** *p* < 0.0001.

It is known that PCV2 replicates in actively dividing cells, since the virus needs host DNA polymerase for its genome replication [20,45]. The in vitro generated T-lymphoblasts exhibited highly proliferative activities, which was induced by a short period of contact with concanavalin A (ConA) and was maintained by a continuous addition of interleukin-2 (IL-2) [30,32,46]. To thoroughly study the dynamics of PCV2 replication in T-lymphoblasts, cells were infected with PCV2 strain 1121 or Stoon1010 at 0.05 TCID_50_/cell for different periods. Cells were fixed and stained for the expression of Rep and Cap proteins, while culture supernatant was collected and titrated by qPCR and virus titration (IPMA). Both Rep and Cap could be detected as early as 12 h for both strains (Figure 2A), with a slightly lower expression of 1121 (0.15% and 0.20%) than Stoon1010 (0.53% and 0.52%). Rep was always found in the nucleus of the cells, being mostly expressed at 24 h in 1.3% and 2.7% of the cells for 1121 and Stoon1010, respectively (Figure 2B). At 24 h, Stoon1010 showed a significantly higher expression of Cap than 1121 did (Figure 2B). The expression of Cap peaked at 36 h for 1121 (1.6%) and at 24 h for Stoon1010 (3.3%), followed by a general decrease of percentages of infected cells until 72 h (Figure 2B). Interestingly, irregularly shaped cells with intense PCV2 antigen and deformed nucleus were found for both strains at 72 h (Figure 2A, white arrow). Virus was released as demonstrated by an increase of viral genome copies in the supernatant from 1.6 × 10^10^ copies at 1 h to 4.7 × 10^11^ copies at 72 h for 1121 and from 2.2 × 10^10^ copies at 1 h to 1.5 × 10^12^ at 72 h for Stoon1010 (Figure 2C). In the second replication cycle, Stoon1010 had a significantly higher amount of genomes released in the supernatant than 1121 had. The titer of extracellular virus for Stoon1010 gradually rose from 10^3.6^ TCID_50_/mL at 1 h to 10^4.6^ TCID_50_/mL at 72 h, while for 1121 the titer slightly increased from 10^2.1^ TCID_50_/mL at 1 h to 10^2.9^ TCID_50_/mL at 12 h and stayed around 10^2.9^ TCID_50_/mL from 12 h till 72 h (Figure 2C). There was a significant difference in virus titers between Stoon1010 and 1121 over time. From these results, we concluded that the in vitro generated T-lymphoblasts supported the replication of PCV2, with strain Stoon1010 replicating more efficiently than strain 1121.

### 3.2. PCV2 Attachment to T-Lymphoblasts is Mediated by Chondroitin Sulfate

#### 3.2.1. PCV2 Attachment is Restricted to 11–26% of T-Lymphoblasts

**Figure 3:**
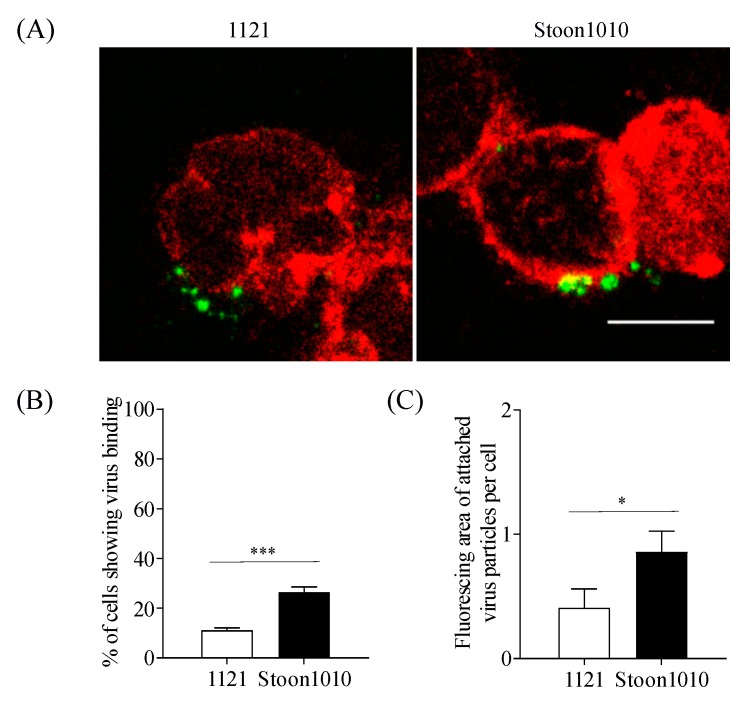
PCV2 attachment to T-lymphoblasts. Cells were incubated with PCV2 particles at 4 °C for 1 h, after which the virus was removed and cells were fixed. PCV2 particles were stained in green, while T-lymphoblasts were stained in red. (**A**) Visualization of PCV2 particles binding to T-lymphoblasts with confocal microscopy. Scale bar = 8 µm. (**B**) The percentage of T-lymphoblasts showing virus binding was determined. (**C**) The fluorescing area of attached virus particles per cell was quantified with ImageJ. Data represent means ± SD of triplicate assays. * *p* < 0.05; *** *p* < 0.001.

Since virus binding is an initial step of the virus infectious cycle, we first examined PCV2 binding to T-lymphoblasts by incubating cells with PCV2 particles at 4 °C for 1 h. The bound virus particles were immunostained and visualized under confocal microscopy. Single and aggregate forms of PCV2 particles of strains 1121 and Stoon1010 bound to 11% and 26% of T-lymphoblasts, respectively (Figure 3A,B). In addition, the amount of attached particles per cell of Stoon1010 was twice that of 1121, as demonstrated by the fluorescence area measured with ImageJ (Figure 3C). These results revealed that PCV2 particles were able to bind certain proportions of T-lymphoblasts, with Stoon1010 being significantly more effective than 1121.

#### 3.2.2. CS, but Not Other GAGs, is Expressed on T-Lymphoblasts

**Figure 4:**
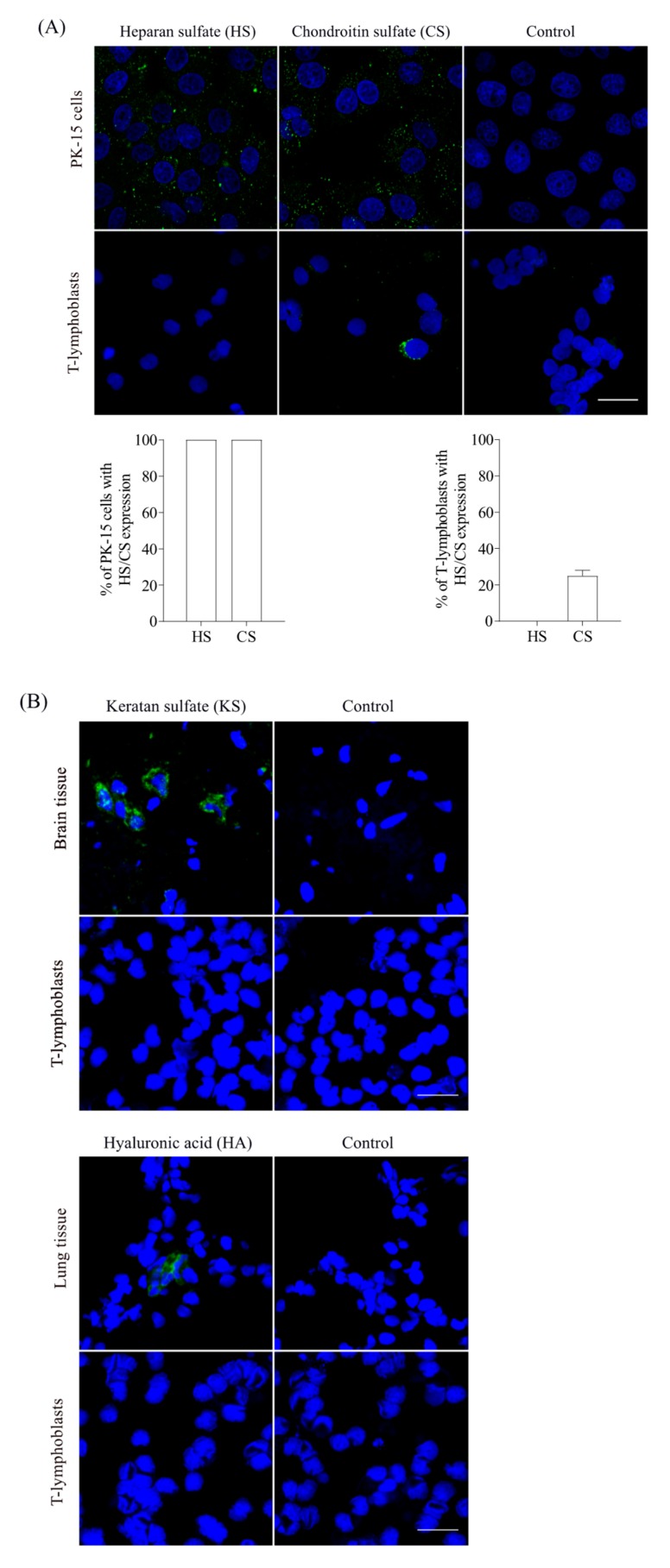
Expression of glycosaminoglycans (GAGs) on T-lymphoblasts. (**A**) Determination of the expression of heparan sulfate (HS) and chondroitin sulfate (CS) on PK-15 cells and T-lymphoblasts. Confocal images show PK-15 cells and T-lymphoblasts staining with specific antibodies against HS and CS. The percentage of PK15 cells/T-lymphoblasts with the expression of HS/CS were calculated and shown. Data represent means ± SD of triplicate assays. (**B**) Confocal images showing T-lymphoblasts and brain/lung tissues which were stained for the expression of keratan sulfate (KS) and hyaluronic acid (HA) using the specific monoclonal antibodies. Scale bar = 25 µm.

Mice thymic T-lymphocytes were found to both biosynthesize and secrete GAGs, consisting of mainly CS and smaller amounts of HS [47]. Here, with specific antibodies, we showed that the expression of HS was not detected on T-lymphoblasts, whereas CS expression was found in 25% of the T-lymphoblasts (Figure 4A). As a positive control, the expression of HS and CS were clearly demonstrated on all PK-15 cells (Figure 4A). Moreover, no expression of KS and HA were found on T-lymphoblasts, whereas their expression was clearly shown in the brain tissue and the lung tissue, respectively (Figure 4B).

#### 3.2.3. PCV2 Particles Were Able to Bind to T-Lymphoblasts With and Without CS Molecules

**Figure 5:**
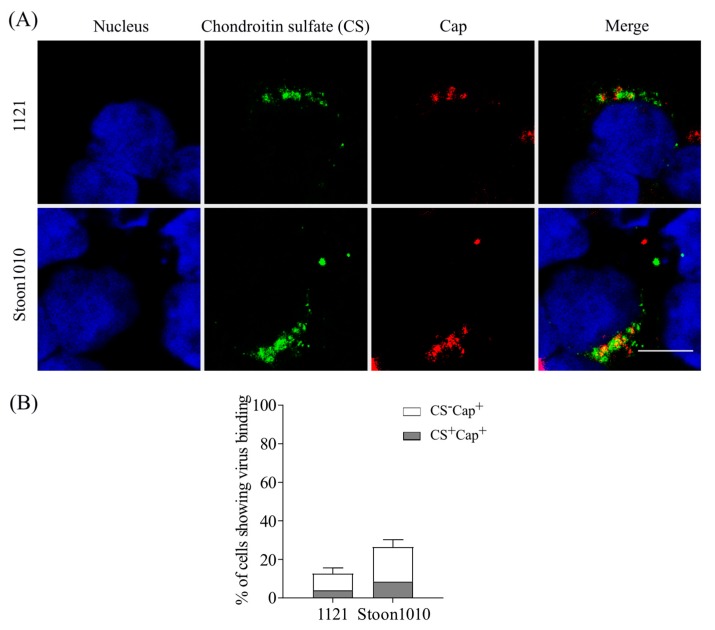
Colocalization of chondroitin sulfate (CS) and PCV2 Cap. T-lymphoblasts were incubated with PCV2 particles at 4 °C for 1 h, after which the virus was removed and cells were fixed. The expression of CS was stained in green, while the bound PCV2 particles were stained in red. Cell nuclei were stained in blue. (**A**) Representative pictures of PCV2 binding to CS-expressed T-lymphoblasts. Scale bar = 8 µm. (**B**) The percentages of T-lymphoblasts showing PCV2 binding and CS expression were calculated. The proportion of cells with bound PCV2 particles and CS expression (CS^+^Cap^+^) was presented in grey, and that with PCV2 binding but not CS expression (CS^−^Cap^+^) in white.

To investigate whether PCV2 attachment to T-lymphoblasts involves CS molecules, the binding assay was performed, after which PCV2 particles and CS molecules were stained with a double immunofluorescence staining assay. PCV2 particles from strain 1121 bound to 12.6% of the total cells, and 3.9% of the total cells (31% of the PCV2+ cells) showing both attached virus particles and CS expression (CS^+^Cap^+^). For strain Stoon1010, 26.3% of T-lymphoblasts showed virus binding, and 8.4% of the total cells (32% of the PCV2+ cells) exhibited bound virus particles as well as CS (CS^+^Cap^+^) (Figure 5). There was a strong colocalization between CS and virus particles. This indicated that only a restricted proportion of PCV2 binding to T-lymphoblasts was mediated by CS.

#### 3.2.4. Enzymatic Removal of CS Decreased PCV2 Infection of T-Lymphoblasts

**Figure 6:**
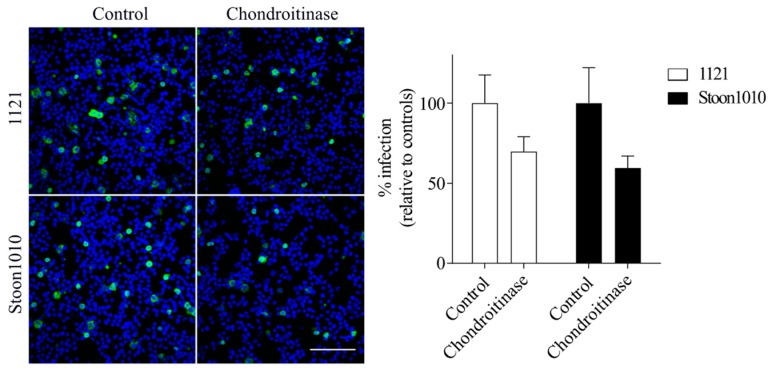
Effect of enzymatic removal of CS on PCV2 infection of T-lymphoblasts. Cells were pre-treated with chondroitinase ABC prior to PCV2 inoculation. The percentage of Cap+ cells (green) was quantified. The infection level in the enzyme-treated group was expressed as the relative percentage to that of the control group (without enzyme treatment). Data represent means ± SD of triplicate assays. Scale bar = 100 µm.

To further determine the role of CS molecules in PCV2 infection to T-lymphoblasts, cells were pre-treated with 2 U/mL chondroitinase ABC prior to inoculation with PCV2 (1121 or Stoon1010). As shown in Figure 6, PCV2 infection in chondroitinase-treated lymphoblasts was reduced by 30% for 1121 and 40% for Stoon1010 compared with non-treated cells. This demonstrated a role of CS in PCV2 attachment to T-lymphoblasts.

### 3.3. PCV2 Entry and Disassembly in T-Lymphoblasts

#### 3.3.1. Visualization of PCV2 Internalization

**Figure 7:**
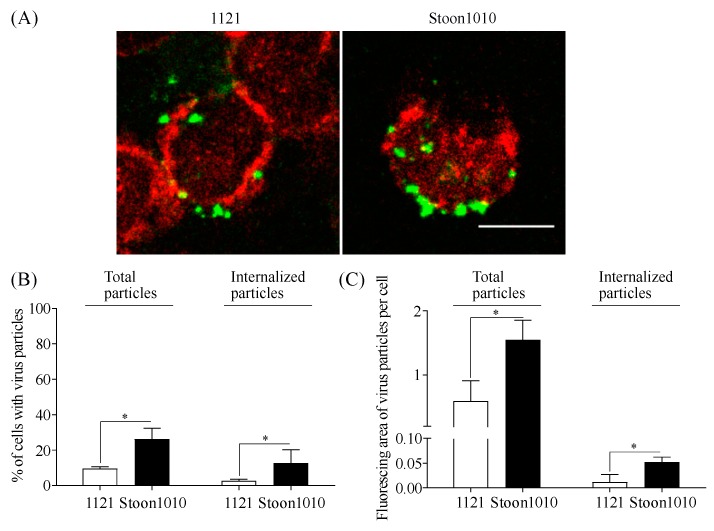
PCV2 internalization into T-lymphoblasts. Cells were incubated with PCV2 particles at 37 °C for 1 h, after which the virus was removed and cells were fixed. PCV2 particles were stained in green, while T-lymphoblasts were stained in red. (**A**) Visualization of PCV2 particles internalized into T-lymphoblasts was performed by confocal microscopy. Some green virus particles were sticking to the cell membrane, while others were found inside the red cell rings and were regarded as internalized particles. Scale bar = 8 µm. (**B**) The percent of T-lymphoblasts with virus (total particles = surface sticking particles + internalized particles) and the percent of T-lymphoblasts with internalized particles was counted. (**C**) The fluorescing area of total virus particles and internalized virus particles per cell was quantified with ImageJ. Data represent means ± SD of triplicate assays. * *p* < 0.05.

Entering the host cell is a crucial step in successful viral infection. Thus, we next examined PCV2 internalization into T-lymphoblasts by incubating cells with PCV2 particles at 37 °C for 1 h. After visualization, 10% and 26% of the cells were respectively found with the immunostained green-fluorescent 1121 and Stoon1010 particles sticking to the cell surface or inside the cells (Figure 7A,B). It turns out that the internalization of PCV2 particles was restricted to 2.6% and 12.7% of the T-lymphoblasts for 1121 and Stoon1010, respectively (Figure 7B). The fluorescing areas of total PCV2 particles (surface-sticking and internalized ones) and of the solely internalized PCV2 particles were quantified with ImageJ. Interestingly, the internalized 1121 and Stoon1010 particles only accounted for 2.0% and 3.4% of the total visualized particles, respectively (Figure 7C). Moreover, a significantly higher amount of Stoon1010 particles were internalized into a larger number of cells than for 1121. These results indicated that during 1 h incubation at 37 °C only a small proportion (2–3.4%) of virus particles entered a restricted number (2.6–12.7%) of T-lymphoblasts, suggesting a slow internalization of PCV2 particles into T-lymphoblasts.

#### 3.3.2. PCV2 Strain 1121, but Not Stoon1010, Exploits Macropinocytosis for Its Entry

**Figure 8:**
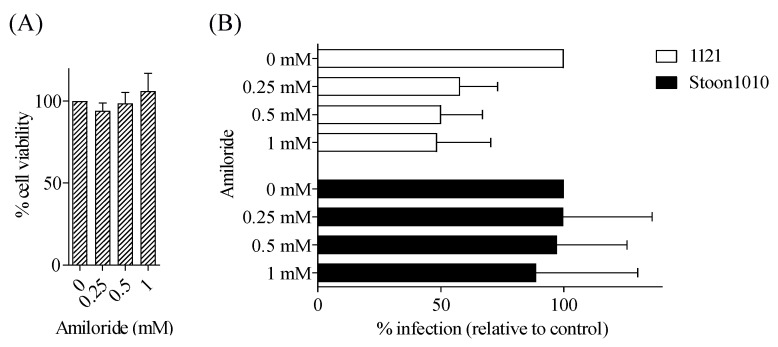
PCV2 strain 1121, but not Stoon1010, exploits macropinocytosis for its entry. (**A**) Viability of cells treated with different concentrations of amiloride, as measured by MTT assay. (**B**) Cells were pre-treated with amiloride (0, 0.25, 0.5, 1 mM) prior to PCV2 infection. The percentage of PCV2-infected cells was quantified. The infection level in the treated group was expressed as the relative percentage to that of the control group (drug concentration = 0 mM). Data represent means ± SD of triplicate assays.

Macropinocytosis has emerged as a major endocytic mechanism which is exploited by over 20 different viruses for their entry into host cells [48]. To test the possible role of macropinocytosis in PCV2 infection, cells were pre-treated with amiloride, a specific inhibitor of macropinocytosis by inhibiting Na^+^/H^+^ exchange. As shown in Figure 8, PCV2 strain 1121 infection was significantly reduced by 42–52% in cells treated with amiloride at concentrations from 0.25 mM to 1 mM. In contrast, PCV2 strain Stoon1010 infection exhibited only an 11% decrease in T-lymphoblasts treated with amiloride at 1 mM compared with untreated cells. This indicated that membrane-bound 1121 may trigger a macropinocytosis pathway for its entry while Stoon1010 does not.

#### 3.3.3. PCV2 Entry Occurs via Clathrin-Mediated Endocytosis and is Independent of Caveolae-Mediated Endocytosis

**Figure 9:**
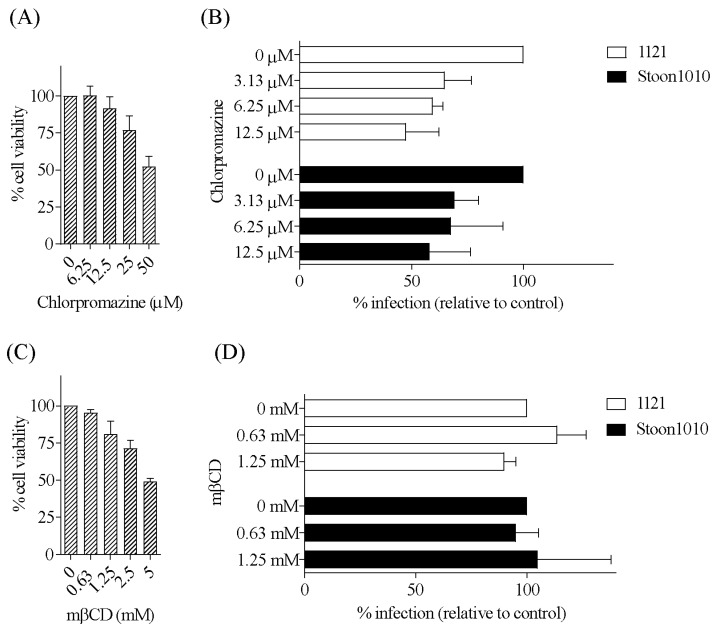
PCV2 entry into T-lymphoblasts is via clathrin-mediated endocytosis, and is independent of caveolae-mediated endocytosis. (**A**,**C**) Viability of cells treated with different concentrations of chlorpromazine (**A**) or mβCD (**C**), as measured by MTT assay. (**B**,**D**) Cells were pre-treated with chlorpromazine (0, 3.13, 6.25, 12.5 µM) (**B**) or with mβCD (0, 0.63, 1.25 mM) (**D**) prior to PCV2 infection. The percentage of PCV2-infected cells was quantified. The infection level in the treated group was expressed as the relative percentage to that of the control group (drug concentration = 0 µM). Data represent means ± SD of triplicate assays.

Non-enveloped viruses are internalized mainly via either clathrin- or caveolae-mediated endocytosis. Thus, the possible role of these pathways in PCV2 entry into T-lymphoblasts was examined. Pre-treatment of T-lymphoblasts with chlorpromazine, a well-known inhibitor of clathrin-mediated endocytosis by affecting the assembly of clathrin lattices at the cell surface and on endosomes, reduced PCV2 infection in a dose-dependent manner and independent of the used PCV2 strain. The maximum reduction of PCV2 infection reached 52% for strain 1121 and 42% for strain Stoon1010, compared with non-treated cells (control) (Figure 9A,B). In contrast, pre-treatment of T-lymphoblasts with mβCD, which inhibits caveolae-mediated endocytosis by removing cholesterol from the plasma membrane, did not result in a significant decrease of PCV2 infection compared with untreated cells. Even at a higher concentration at 1.25 mM, this compound did not show any effect on PCV2 infection (Figure 9C,D). Taken together, these results suggested that PCV2 entry into T-lymphoblasts required clathrin but not caveolae.

#### 3.3.4. PCV2 Entry Into T-Lymphoblasts Requires Actin

**Figure 10:**
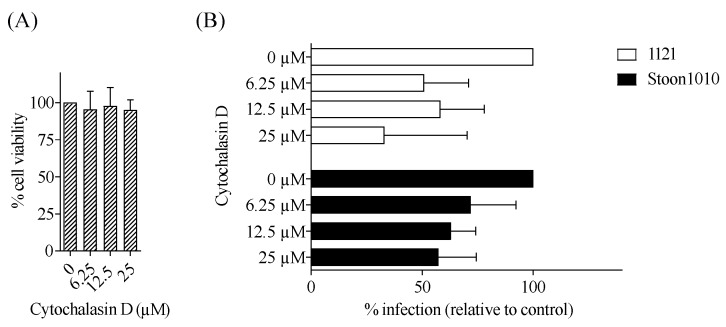
PCV2 entry into T-lymphoblasts requires actin. (**A**) Viability of cells treated with different concentrations of cytochalasin D, as measured by MTT assay. (**B**) Cells were pre-treated with cytochalasin D (0, 6.25, 12.5, 25 µM) prior to PCV2 infection. The percentage of PCV2-infected cells was quantified. The infection level in the treated group was expressed as the relative percentage to that of the control group (drug concentration = 0 µM). Data represent means ± SD of triplicate assays.

The function of actin cytoskeleton in clathrin-mediated endocytosis has been suggested [49]. In addition, previous studies showed that actin is required in PCV2 infection of 3D4/31 cells, epithelial cells and primary porcine monocytes [25,27,29]. To test whether actin is involved in PCV2 infection in T-lymphoblasts, cells were pre-treated with cytochalasin D, an inhibitor of actin polymerization. As a result, treatment of T-lymphoblasts with increased concentrations of this inhibitor caused a decreased PCV2 infection in a dose-dependent fashion, to a maximum of 67% reduction for strain 1121 and 43% for Stoon1010 at 25 µM (Figure 10). These results confirmed the requirement of actin in PCV2 entry into T-lymphoblasts.

#### 3.3.5. Participation of Small GTPases in PCV2 Entry into T-Lymphoblasts

**Figure 11:**
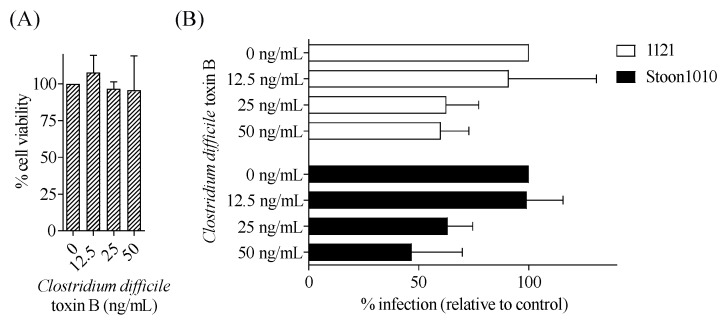
Participation of small GTPases in PCV2 entry into T-lymphoblasts. (**A**) Viability of cells treated with different concentrations of *Clostridium difficile* toxin B, as measured by MTT assay. (**B**) Cells were pre-treated with *Clostridium difficile* toxin B (0, 12.5, 25, 50 ng/mL) prior to PCV2 infection. The percentage of PCV2-infected cells was quantified. The infection level in the treated group was expressed as the relative percentage to that of the control group (drug concentration = 0 ng/mL). Data represent means ± SD of triplicate assays.

The Rho GTPases are key regulators of the actin cytoskeleton and are involved in macropinocytosis, clathrin-mediated endocytosis and clathrin- and caveolae-independent internalization pathways [50]. To investigate the role of small GTPase on PCV2 infection, T-lymphoblasts were treated with a broad range small GTPase inhibitor, *Clostridium difficile* toxin B. Consequently, a dose-dependent decrease of PCV2 infection in T-lymphoblasts was found, with 40% and 53% reduction by the inhibitor at 50 ng/mL for strain 1121 and Stoon1010, respectively (Figure 11). This suggested a role of small GTPases in PCV2 entry into T-lymphoblasts.

#### 3.3.6. PCV2 Infection of T-Lymphoblasts Requires a Low-pH Step

**Figure 12:**
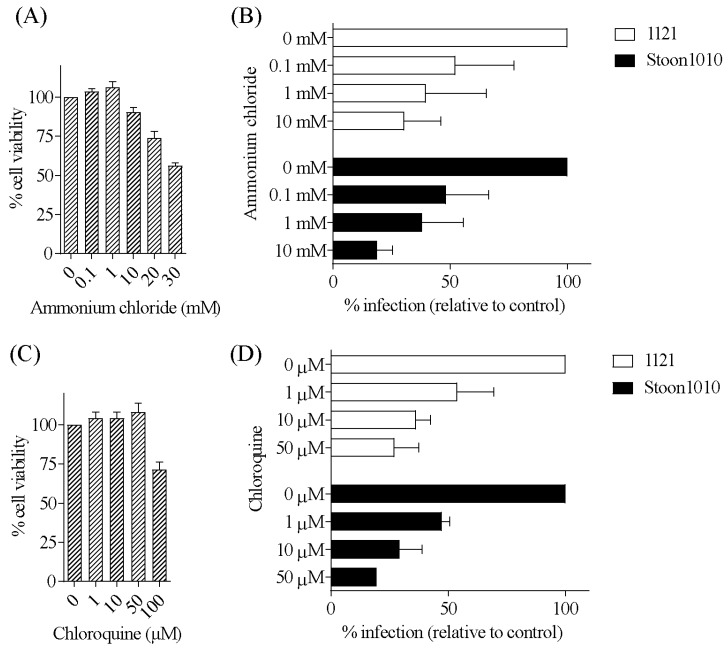
PCV2 infection of T-lymphoblasts requires a low-pH step. (**A**,**C**) Viability of cells treated with different concentrations of ammonium chloride (**A**) or chloroquine (**C**), as measured by MTT assay. (**B**,**D**) Cells were pre-treated with ammonium chloride (0, 0.1, 1, 10 mM) (**B**) or with chloroquine (0, 1, 10, 50 µM) (**D**) prior to PCV2 infection. The percentage of PCV2-infected cells was quantified. The infection level in the treated group was expressed as the relative percentage to that of the control group (drug concentration = 0 mM). Data represent means ± SD of triplicate assays.

Viruses that enter via clathrin-mediated endocytosis are transported to the endosome-lysosome system where the incoming viruses are exposed to a pH drop [51]. To determine whether a pH drop was a crucial step in the infectious entry of PCV2 into T-lymphoblasts, we used two lysosomotropic weak bases (ammonium chloride and chloroquine diphosphate) to raise the endosomal pH and examined the effect on PCV2 infection. Pre-treatment of T-lymphoblasts with increased concentrations of these inhibitors resulted in a dose-dependent significant decrease of PCV2 infection compared with non-treated cells, with a maximum reduction of 70–81% by ammonium chloride at 10 mM (Figure 12A,B) and of 73–81% by chloroquine diphosphate at 50 µM (Figure 12C,D). Taken together, these results showed that PCV2 entered T-lymphoblasts in a low-pH-dependent manner.

#### 3.3.7. PCV2 Disassembly is Mediated by Serine Proteases

**Figure 13:**
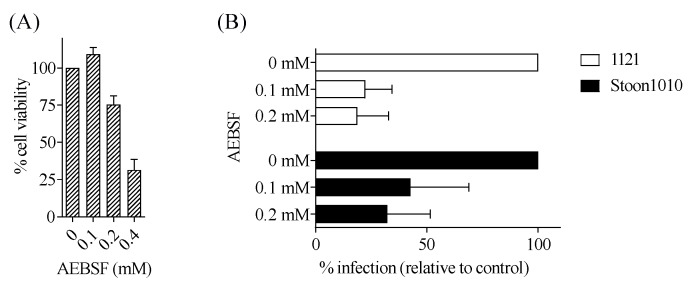
PCV2 disassembly in T-lymphoblasts is mediated by serine proteinases. (**A**) Viability of cells treated with different concentrations of AEBSF, as measured by MTT assay. (**B**) Cells were pre-treated with AEBSF (0, 0.1, 0.2 mM) prior to PCV2 infection. The percentage of PCV2-infected cells was quantified. The infection level in the treated group was expressed as the relative percentage to that of the control group (drug concentration = 0 mM). Data represent means ± SD of triplicate assays.

Virus disassembly within the endosomal–lysosomal system can be mediated by cellular proteases, which are classified into serine, cysteine, aspartic and metalloproteases [52]. Serine proteases mediate the disassembly of PCV2 in 3D4/31 monocytic cells and PK-15 cells [53]. To investigate the role of serine proteases in PCV2 infection to T-lymphoblasts, cells were treated with the serine protease inhibitor AEBSF at different concentrations prior to PCV2 inoculation. The results showed that 68–81% of PCV2 infection was abolished by AEBSF at 0.25 mM in the treated group compared with the untreated group (Figure 13).

### 3.4. Structural Difference of PCV2 Cap between Strains 1121 and Stoon1010

**Figure 14:**
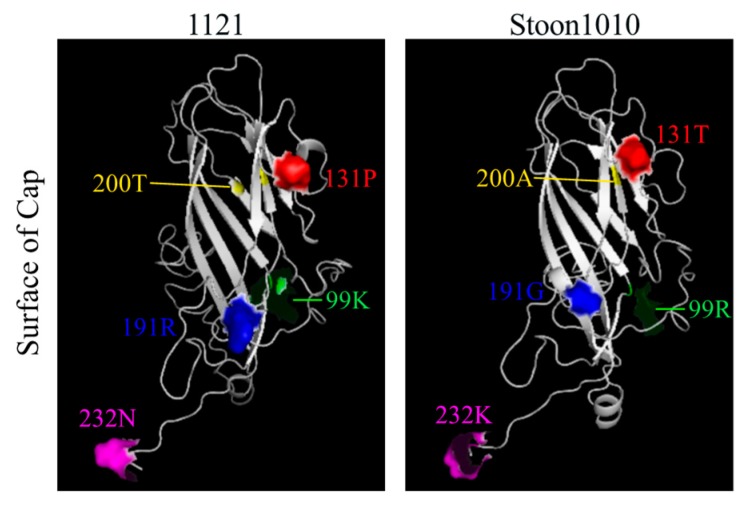
Location of strain-specific amino acids on the respective ribbon diagram of the PCV2 capsid protein of strains 1121 and Stoon1010. The 3D structures of the capsids were generated using I-TASSER server and displayed with PyMOL.

Alignment of the amino acid sequences from strains 1121 and Stoon1010 revealed different amino acids at five positions: 99K (1121)/R (Stoon1010), 131P/T, 191R/G, 200T/A and 232N/K. These amino acids were mapped on the 3D structure of the Cap protein (Figure 14). The amino acid K (1121)/R (Stoon1010) at position 99 is located at the inner side of the Cap protein, and is relatively conserved, since K and R are both basic polar amino acids. Proline (P) has a special cyclic structure, which contributes to the rigidity of the structure. Mutations from a non-polar amino acid (P) in 1121 to a polar neutral amino acid (T) in Stoon1010 at position 131 may lead to a more flexible protein structure for stoon1010 than 1121. Similarly, at position 191, a basic polar amino acid (R) is replaced by glycine (G) that is more flexible than other amino acids; at position 200, a polar neutral amino acid (T) mutates to a non-polar hydrophobic one (A). At position 232, a polar neutral amino acid N in 1121 is replaced by a basic polar amino acid K in Stoon1010, which makes the virion of Stoon1010 more positively charged than that of 1121. This indicates a possible structurally conformational difference in the Cap protein of strains 1121 and Stoon1010. 

**Figure 15:**
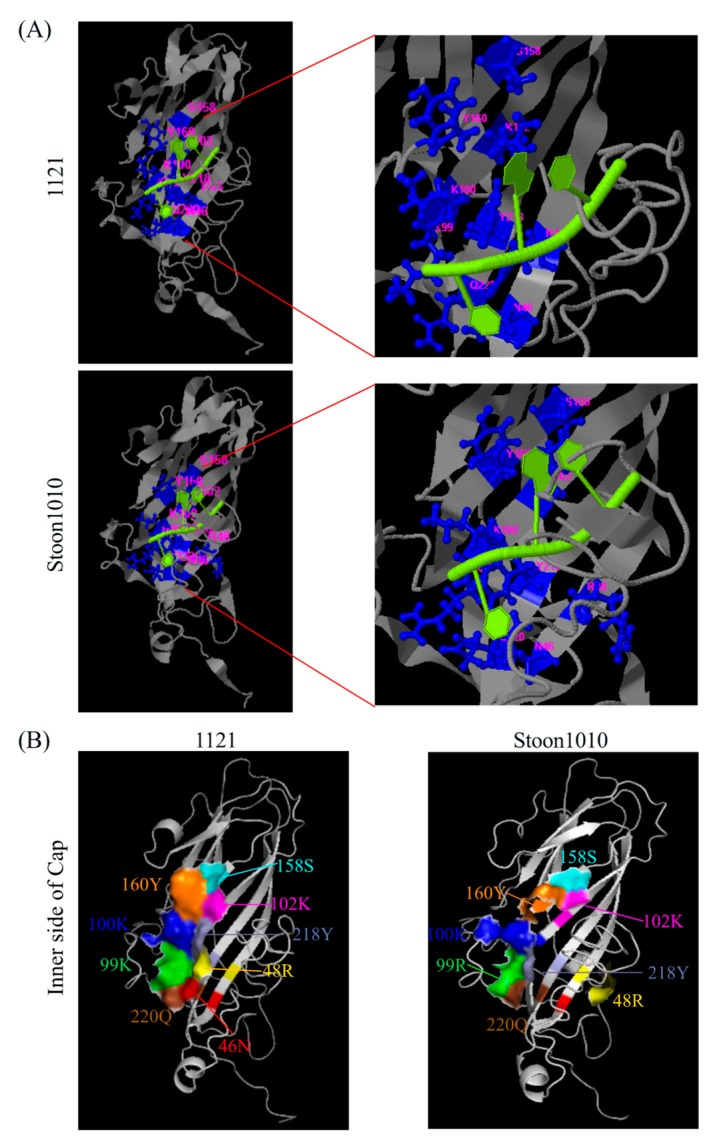
Binding PCV2 Cap protein to the nucleic acid. (**A**) Predicted models of PCV2 Cap protein binding to nucleic acid. Ribbon diagram of the PCV2 capsid protein are shown with a full view (left) and a zoomed view (right). Binding residues are shown in blue balls and sticks, and are labelled with magenta; predicted binding nucleic acid is shown in green/yellow. (**B**) Location of amino acids that are at the predicted binding sites. Different color codes were used for different amino acid sites, and the same color was used for the same amino acid site across strains 1121 and Stoon1010. The 3D structures of capsid were generated using I-TASSER server and displayed with PyMOL.

The conformational changes in Cap protein may affect its ability to interact with viral nucleic acid for virion assembly. Based on the 3D structure of PCV2 Cap of strains 1121 and Stoon1010, the nucleic binding sites of the Cap protein were predicted to be at positions 46N, 48R, 99K/R, 100K, 102K, 158S, 160Y, 218Y and 220Q (Figure 15A). These amino acids were mapped on the 3D structure of the Cap protein, and they are at the inner part of the capsid (Figure 15B). For strain 1121, the surface areas of these amino acids are very close to each other and form a large surface area; while for strain 1010, the surface areas are dispersed into three parts: one part is formed by amino acids at positions 160Y, 158S and 102K; another one consists of amino acids at positions 100K, 99R, 220Q and 218Y; the last one is the amino acid at 48R, and the amino acid at position 46N does not display its surface anymore. These changes most probably result from the conformational changes which are induced by strain-specific amino acid mutations, leading to the different affinity of the Cap protein to viral nucleic acid.

## 4. Discussion

PCV2 is one of the most economically important pig pathogens, causing PCV2-associated diseases in most major pig producing countries. Currently, these diseases are well controlled by vaccination. However, vaccination does not lead to a sterile immunity [54]. Since viral entry is an attractive target for therapeutic intervention, it is imperative that we understand the mechanism of PCV2 entry and replication in its natural target cells. PCV2 mainly targets at lymphoblasts and monocytic cells. Primary monocytes and dendritic cells take up virus particles via clathrin-mediated endocytosis, but this rarely leads to a productive viral infection [26,27]. In contrast, lymphoblasts are fully susceptible targets [19,30]. The larger the number of blasts, the faster and higher the primary replication of PCV2 in its host [31]. However, the PCV2 replication cycle in lymphoblasts was not elucidated.

In the present study, we generated proliferative porcine T-lymphoblasts in vitro and demonstrated that these cells supported PCV2 replication. The initial virus binding was mediated by chondroitin sulfate for 11% (1121) and 26% (Stoon1010) of T-lymphoblasts, after which the bound virus particles were internalized into a restricted proportion of the cells (2.6% (1121) and 12.7% (Stoon1010)) via clathrin-mediated endocytosis with the participation of actin and small GTPases. These internalized virus particles were then transported to the endosome-lysosome system and disassembled in an acid environment aided by serine proteases. Furthermore, PCV2 strain Stoon1010 replicated better than 1121. This was due to a more efficient virus binding and internalization in a higher proportion of T-lymphoblasts for Stoon1010 than for 1121, despite the fact that strain 1121 could exploit macropinocytosis as well as an alternative way of entry. This was indicative of a biological difference between PCV2 strains in the replication in porcine T-lymphoblasts.

It is known that PCV2 replication is highly dependent on cell mitosis since it needs the host DNA polymerase for its genome replication. Therefore, PK-15 cells and swine kidney (SK) cells have been routinely used for PCV2 research purposes. However, these cells are not PCV2 target cells in vivo whereas lymphoblasts are. The in vitro generation of porcine T-lymphoblasts allowed us to get closer to the real in vivo situation to study PCV2 pathogenesis. Recent research demonstrated that the in vitro generated T-lymphoblasts supported a productive replication of PCV2 as found in vivo [19,31,32]. The mechanism of PCV2 replication was examined in detail in the present study. The expression kinetics of the Rep and Cap proteins were established. Rep and Cap proteins were detected as early as 12 h. The percentage of cells with Rep expression peaked at 24 h and dropped until 72 h, due to the continuous proliferation of cells and at the end due to the lysis of PCV2-infected cells (Figure 2A, white arrow). Rep was exclusively found in the nucleus of T-lymphoblasts except in the lysed cells, which was the same as in PK-15 cells [55]. At 12 h, Cap could already be detected in the nucleus of the cells. This is in line with previous studies on PCV2 replication in L35 lymphoblastoid cells and in PK-15 cells [55,56]. The presence of Cap in the nucleus at 12 h suggested an earlier initial synthesis in the cytoplasm, probably around 6 h, as demonstrated in PK-15 cells [55]. Later on, strains 1121 and Stoon1010 exhibited different replication characteristics, with the Cap expression of 1121 peaking at 36 h and Stoon1010 at 24 h. Afterwards, the percentage of Cap+ cells did not further increase as a result of a second replication cycle. This is not only due to the lysis of PCV2-infected cells (Figure 2A, white arrow), but also due to a contact inhibition of cell proliferation.

Replication of strain 1121 in T-lymphoblasts is less efficient than that of Stoon1010, which was reflected by a lower expression of antigen-positive cells, a lower virus titer and a smaller number of virus genome copies in the supernatant. For Stoon1010, the progeny virus was released between 24 h and 36 h, as revealed by a fast increase of viral genome copies and virus titers in the supernatant. Interestingly for 1121, although a logarithmic increase of the number of viral genomes in the supernatant was demonstrated, the virus titer stayed at 10^2.9^ TCID_50_/mL from 12 h to 72 h. It seems that strain 1121 had problems with viral genome encapsidation, which resulted in an inefficiency in virion assembly and release. Virion assembly is driven by the interaction between the viral capsid and the viral genome. The binding sites of nucleic acids to the Cap protein from strains 1121 and Stoon1010 was predicted to be amino acids at positions 46N, 48R, 99K/R, 100K, 102K, 158S, 160Y, 218Y and 220Q (Figure 15A). These amino acids are conserved among these two strains, despite the fact that at position 99 a basic polar amino acid (K) in 1121 is replaced with another basic polar one (R) in Stoon1010. Interestingly, although amino acids in the binding sites are conserved, the Cap protein of 1121 showed a lower affinity and stability to the nucleic acid compared with Stoon1010 (Figure 15A). When these amino acids at the binding sites were mapped on the respective 3D structure of the Cap protein from 1121 and Stoon1010, they displayed different surface patterns. Those from 1121 were very close to each other, whereas those from Stoon1010 were dispersed (Figure 15B). This structural difference is probably the result of conformational changes that are induced by strain-specific amino acids of 1121/Stoon1010: 99K/R, 131P/T, 191R/G, 200T/A and 232N/K (Figure 15). For instance, proline (P) has a distinctive cyclic structure, which contributes to the rigidity of the structure. The mutation from a non-polar amino acid (P) in 1121 to a polar neutral amino acid (T) in Stoon1010 at position 131 may lead to a more flexible protein structure for stoon1010 than 1121. Similarly, at position 191, a basic polar amino acid (R) is replaced by glycine (G) that is more flexible than other amino acids; at position 200, a polar neutral amino acid (T) mutated to a non-polar hydrophobic one (A). The more flexible and loose structure of the Cap protein from Stoon1010 compared to that of 1121 may allow a better interaction of the capsid with the viral genome, and therefore a more efficient virion assembly. In line with our findings, Fenaux et al. (2004) demonstrated that proline at positions 110 and arginine at position 191 influenced the production of infectious virus [57]. He found when the wild type virus (VP1) was passaged 120 times in PK-15 cells (VP120), VP120 replicated more efficiently in PK-15 cells than VP1. Differences between VP1 and VP120 were a mutation from proline (P) to alanine (A) at position 110 (P110A) and a mutation from arginine (R) to serine (S) at position 191 (R191S) [57]. Taken together, this may suggest that proline (P) at positions 110/131 and arginine (R) at position 191 are able to reduce PCV2 infection, by affecting PCV2 virion assembly and thus the production of infectious virus.

Virus binding is the first step to initiate a successful infection of cells. PCV2 attachment to T-lymphoblasts was restricted to 11–26% of the cells, which was apparently less efficient compared with 3D4/31 monocytic cells and the epithelial cell lines PK-15, ST and SK where all the cells showed virus binding [25,29]. This high efficiency of PCV2 attachment to these cells were mediated by HS and CS [24]. Interestingly, in contrast to the strong expression of these two molecules on all PK-15 cells, none of the T-lymphoblasts showed the expression of heparan sulfate while only 25% of the cells expressed chondroitin sulfate. In addition, no expression of KS and HA was found. Indeed, leukocytes are known to express GAGs primarily of the CS type. In agreement with our finding, little or no cell surface HS could be detected on human mitogen-activated human T cells [47,58]. We hypothesized that the limited expression of CS on T-lymphoblasts accounted for the restricted binding of PCV2 to these cells. This hypothesis was supported by the result that PCV2 infection to T-lymphoblasts was decreased (30–40% of reduction) after the enzymatic removal of chondroitin sulfate with chondroitinase ABC. The reduced yet non-abolished PCV2 infection may be explained by the fact that not all PCV2-bound cells showed the expression of CS molecules. Despite the fact that around 11–26% of cells exhibited virus binding, only 4–8% of cells showed CS expression as well as bound virus particles, accounting for around 30% of the virus-bound cells. This indicates that 70% of the virus binding is mediated by other molecules. In line with our finding, previous research revealed that PCV2 could still infect mutant Chinese hamster ovary (CHO) cells which do not express GAGs [24]. These results indicate that, apart from the two identified PCV2 attachment GAG receptors, other yet unidentified cellular surface molecules participate in virus binding. The difference in the expression of HS and CS in PK-15 cells and T-lymphoblasts could partly explain the distinct characteristics of PCV2 binding to these two cell types. Virus particles of Stoon1010 showed a more efficient binding to T-lymphoblasts than those of 1121. Interestingly, the polar neutral amino acid N at position 232 in 1121 was replaced by a basic polar amino acid K in Stoon1010 (Figure 15). Since position 232 is at the C-termini of the Cap protein, the amino acid at this position is exposed and extended on the virion surface [59]. Therefore, the basic polar amino acid K on the virion surface of Stoon1010 may enhance the interaction of virions with the cell membrane-associated negatively charged chondroitin sulfate, thus enhancing virus binding. Of interest, the most recent dominant PCV2 genotype PCV2d also has an extra basic polar amino acid K at position 234, which is possibly related with the increased virulence of strains of this genotype than the previous PCV2a and PCV2b [60,61]. It is very well possible that there was an evolutionary change from 1121 to Stoon1010, by exposing more positively charged amino acids on the virion surface for a better binding and survival in animal cells.

Following binding to cell surface receptors, viruses must be internalized to initiate infection. In this study, internalization of PCV2 into T-lymphoblasts was restricted to 2.6–12.7% (1121-Stoon1010) of total cells and 2.0–3.4% (1121-Stoon1010) of the cells internalized virus particles after 1 h incubation at 37 °C. This indicated a slow and inefficient entry of PCV2 into T-lymphoblasts, which may result in the long replication cycle (24–36 h) of PCV2 in susceptible cells [25]. In general, viruses accomplish cell entry by hijacking the cellular endocytic machinery. Thus, we further defined the endocytic mechanisms of PCV2 entry into T-lymphoblasts using pharmacological inhibitors. We demonstrated that PCV2 uses clathrin-mediated endocytosis to enter T-lymphoblasts and this entry was dependent on actin and small GTPases. This is somewhat the same with the mechanism of PCV2 entry into the monocytic cell line 3D4/31, primary monocytes and dendritic cells where the clathrin-mediated endocytosis was used [25,26,27]. Despite this similarity, the clathrin-mediated pathway was an ineffective one in the epithelial cell lines PK15, SK and ST, since blocking this pathway with chlorpromazine increased PCV2 infection in these cells [29]. Instead, PCV2 exploited a dynamin-and cholesterol-independent, but actin- and small GTPase-dependent pathway for its entry into the epithelial cell lines [29]. This means that PCV2 is able to employ multiple entry modes depending on the cell type. Likewise, the existence of alternative pathways for internalization into different cell types has been reported for reovirus [62], human immunodeficiency virus (HIV) [63], equine herpes virus (EHV) [64], herpes simplex virus (HSV) [65] and Japanese encephalitis virus (JEV) [66,67].

Interesting to note is that we demonstrated for the first time that PCV2 entry modes were strain-dependent. Apart from the classical clathrin-mediated endocytosis, PCV2 strain 1121 could exploit macropinocytosis as well for its entry into T-lymphoblasts while strain Stoon1010 could not do this. This was based on the result that amiloride, the specific inhibitor of marcopinocytosis, blocked 52% of PCV2 strain 1121 infection to T-lymphoblasts but had little effect on the infection of strain Stoon1010 (11% of reduction). Apparently, PCV2 belongs to a growing group of viruses that seem to make use of different, alternative strategies and pathways to enter cells. Influenza virus is another well-studied example of this; it can exploit clathrin-endocytosis and macropinocytosis simultaneously in HeLa, A549 and other non-human cells [68]. Similarly, Ebola virus particles entry into HeLa cells mainly occurs via macropinocytosis, but a smaller fraction of particles enters via clathrin-mediated endocytosis [69]. Probably, viruses have evolved to exploit different cellular pathways in order to increase their host range. However, in this case, for PCV2 strain 1121, this wide use of internalization options is not an advantage due to the fact that the internalization of this strain (2.6% of the cells) was less efficient than that of Stoon1010 (12.7% of the cells). This again reflects an evolution of PCV2 from 1121 to Stoon1010, from using non-specific macropinocytosis pathway for entry to the more specific and efficient receptor-mediated entry pathway. Especially when one considers the fact that animal circoviruses may be derived from plant nanoviruses that may have infected a vertebrate host [70], the entry of circovirus into animal cells at the very beginning could take advantage of the natural and non-specific cellular macropinocytosis (cell drinking). With the gradual adaption of circovirus to animal cells, it may evolve to find its specific and efficient way for entry. It will be very interesting to study which amino acid mutation(s) of 1121 and Stoon1010 led to the shift in the endocytic pathway. Both strains 1121 and Stoon1010 belong to the PCV2a genotype. Since PCV2 shifts from PCV2a to PCV2b and later on to PCV2d [71,72], it would be interesting to examine the entry pathways of PCV2b and PCV2d strains, to see whether PCV2b and PCV2d strains during evolution use more efficient entry modes and whether the increased viral virulence results from a more efficient entry into host cells. This is the first study where we examined the entry features of strain 1121 which was isolated from an aborted fetus [34], since strain Stoon1010 which was isolated from a PMWS piglet was always used in all previous entry studies [25,29,33]. Previous work from Lefebvre et al. demonstrated that these two PCV2 strains have different neutralizing epitopes since the mAbs that neutralized Stoon1010 did not differentiate this strain from 1121 in the IPMA [37]. Based on this result, they suggested that these two PCV2 strains might use different entry pathways in susceptible cells and this hypothesis was confirmed in our study. It will be interesting as well to study whether these different entry modes of PCV2 strains to the target cells can affect the final outcome of PCV2 infections. It is worthwhile to mention that a combination of amiloride (1 mM, inhibitor of macropinocytosis) and chlorpromazine (6.25 µM or 12.5 µM, inhibitor of clathrin-mediated endocytosis) cannot fully block PCV2 entry/infection to T-lymphoblasts (Figure S1), indicating that other yet unidentified pathways or mechanisms exist and account for the residual infection.

Following virus internalization, the incoming virions need to be uncoated, after which the viral genome becomes released and transported to the appropriate sites for replication. Just like the internalization, the disassembly process of PCV2 fully depends on the host cells used. In the monocytic cell line 3D4/31, PCV2 needs an acid environment for virion disassembly [25], whereas in the epithelial cell lines PK15, SK and ST, blocking the pH drop enhances PCV2 replication [53]. In this study, we demonstrated that PCV2 replication in T-lymphoblasts required a pH drop in the endosome. By using the protease inhibitor AEBSF, we showed that serine proteases are involved in the disassembly of PCV2 in T-lymphoblasts, as also suggested in the 3D4/31 cells and the epithelial cell lines [53]. Hence, we believe that different serine proteases which are active at a low pH (in the 3D4/31 cells and T-lymphoblasts) and at a neutral pH (in the epithelial cell lines) are involved in PCV2 disassembly. Further work needs to be done to identify these serine proteases.

In summary, we demonstrated that the in vitro generated porcine T-lymphoblasts support PCV2 replication. Virus binding was partially mediated by CS in a restricted population of T-lymphoblasts. Only a small fraction of the bound virus particles was internalized via clathrin-mediated endocytosis which was dependent on actin and small GTPases. The incoming virus required an acid environment for virion disassembly under the function of serine proteases. Furthermore, we revealed for the first time that PCV2 strain 1121 but not Stoon1010 was able to exploit macropinocytosis as an additional entry pathway apart from the classical clathrin-mediated one. We hypothesize an evolution from 1121 to Stoon1010, as revealed by (i) an enhanced virion binding to cells; (ii) a more specific receptor-mediated entry into cells; and (iii) an increased affinity of viral capsids to viral nucleic acids of Stoon1010 compared to 1121. Site-directed mutagenesis on a PCV2 infectious clone will be performed in the future to investigate which amino acid(s) could be responsible for the difference in virus binding, entry and virion assembly. Further unravelling of biological differences in-between PCV2 strains among different genotypes may provide new insights into the evolutionary changes of PCV2 in order to improve its replication in its target cells and the complex pathogenesis of PCVAD.

## Supplementary Materials

The following are available online at https://www.mdpi.com/1999-4915/11/9/813/s1, Figure S1: Effect of a combination of amiloride and chlorpromazine on PCV2 entry/infection of T-lymphoblasts.

## Abbreviations

AEBSF	4-(2-aminoethyl) benzenesulfonyl fluoride
ATCC	American Type Culture Collection
Cap	capsid protein
CHO	Chinese hamster ovary
ConA	concanavalin A
CS	chondroitin sulfate
DABCO	1,4-diazobicyclo-2.2.2-octane
DPBS	Dulbecco’s PBS
DSHB	the Developmental Studies Hybridoma Bank
FCS	fetal calf serum
GAG	glycosaminoglycan
HA	hyaluronic acid
HIV	human immunodeficiency virus
HS	heparan sulfate
HSV	herpes simplex virus
IL-2	interleukin-2
IPMA	immunoperoxidase monolayer assay
I-TASSER	iterative threading assembly refinement
JEV	Japanese encephalitis virus
KS	keratan sulfate
mAb	monoclonal antibody
mβCD	methyl-β-cyclodextrin
MTT	metabolize 3-(4,5-dimethylthiazol-2-yl)-2,5-diphenyltetrazolium bromide
ORF	open reading frame
PBMC	porcine blood mononuclear cell
PCV	porcine circovirus
PCVAD	PCV2-associated disease
PF	paraformaldehyde
PMWS	postweaning multisystemic wasting syndrome
Rep	replicase protein
RT	room temperature
SK	swine kidney
