# Supplementary material for: Strain-Dependent Porcine Circovirus Type 2 (PCV2) Entry and Replication in T-Lymphoblasts
Source: Viruses. 2019 Sep 2;11(9):813. doi: 10.3390/v11090813 (PMC6783876; doi:10.3390/v11090813)
Supplement: Supplementary file 1 [file viruses-11-00813-s001.zip › viruses-578206-suppl.docx]

Figure S1: Effect of a combination of amiloride and chlorpromazine on PCV2 entry/infection of T-lymphoblasts.

Figure S1. Dual inhibition of macropinocytosis and clathrin-mediated endocytosis cannot fully block PCV2 entry/infection to T-lymphoblasts. Cells were pre-treated with a mixture of amiloride (1mM) and chlorpromazine (6.25 µM or 12.5 µM) prior to PCV2 infection. The percentage of PCV2-infected cells was quantified. The infection level in the treated group was expressed as the relative percentage to that of the control group (drug concentration = 0 mM). Data represent means ± SD of triplicate assays. Combination of amiloride and chlorpromazine cannot fully block PCV2 entry/infection to T-lymphoblasts, indicating that other yet unidentified pathways or mechanisms exist and account for the residual infection.
